# Spontaneous Voice Gender Imitation Abilities in Adult Speakers

**DOI:** 10.1371/journal.pone.0031353

**Published:** 2012-02-17

**Authors:** Valentina Cartei, Heidi Wind Cowles, David Reby

**Affiliations:** 1 School of Psychology, Sussex University, Brighton, United Kingdom; 2 Department of Linguistics, University of Florida, Gainesville, Florida, United States of America; University of Saint-Etienne, France

## Abstract

**Background:**

The frequency components of the human voice play a major role in signalling the gender of the speaker. A voice imitation study was conducted to investigate individuals' ability to make behavioural adjustments to fundamental frequency (F0), and formants (Fi) in order to manipulate their expression of voice gender.

**Methodology/Principal Findings:**

Thirty-two native British-English adult speakers were asked to read out loud different types of text (words, sentence, passage) using their normal voice and then while sounding as ‘masculine’ and ‘feminine’ as possible. Overall, the results show that both men and women raised their F0 and Fi when feminising their voice, and lowered their F0 and Fi when masculinising their voice.

**Conclusions/Significance:**

These observations suggest that adult speakers are capable of spontaneous glottal and vocal tract length adjustments to express masculinity and femininity in their voice. These results point to a “gender code”, where speakers make a conventionalized use of the existing sex dimorphism to vary the expression of their gender and gender-related attributes.

## Introduction

The human voice is highly sexually dimorphic. Alongside other properties that distinguish male from female voices, such as intonation [1], duration [2], [3] and speech rate [4], [5], the main cues to speaker gender are fundamental frequency (F0 - or its perceptual correlate “pitch”) and formant frequencies (Fi - mainly responsible for the perception of “timbre”), which together account for 98.8% of the perceived voice dimorphism [6].

These differences stem from the testosterone-driven enlargement of the larynx and the increase in the length of the vocal tract that accompany male puberty [7]. During this time, the male larynx outgrows the female larynx by 40% [7], increasing vocal fold length by 60% on average (reaching 16 mm in adult males, and 10 mm in adult females [8]). As F0 is based on the rate of vocal fold vibration, which in turn is inversely proportional to the square root of the vocal fold tissue length, men's F0 (about 120 Hz) becomes on average 80 Hz lower than women's (about 200 Hz) [7] giving male speakers their characteristically lower-pitched voice. Between-sex differences in formant frequencies are related to differential body growth, with adult men being 7% taller than women on average [9] and to the male-specific second descent of the larynx, which together contribute to men's vocal tract being on average 18 cm, compared to women's 15 cm [10]. Because formant frequencies are negatively correlated with the length of the vocal tract [11], male speakers produce lower Fi values and therefore a formant spacing (ΔF) that is about 15%–20% narrower than in female speakers [12], [13], which results in male voices having a more “baritone” timbre [14].

Variation in gender expression, however, cannot be entirely determined by these hormonal and size-related sex differences in the vocal apparatus. For example, acoustic analyses [15]–[19] of pre-pubertal children's voices consistently show that boys speak with lower formants than girls, while perceptual studies [18] show that children's voice gender can be identified in children as young as 4 years old, despite the fact that the anatomy of the vocal apparatus does not significantly differ between the two sexes until the pubertal age [14], [20]. These observations suggest that children acquire (consciously or unconsciously) gender-specific articulatory behaviours during development, and that speakers develop a knowledge of how a “male” or a “female” should sound, with male voices being low-pitched and “deeper”, while female voices being high-pitched and “lighter”. These differences in formant frequencies also suggest a possible role for lip protrusion (or spreading) and larynx lowering (or raising) in vocal tract length adjustments during speech, as possible articulatory gestures used by speakers in order to masculinise or feminise their voices. Thus, on top of the static, bio-hormonally determined differences, our voice contains dynamic and behaviourally controlled acoustic cues (in particular F0 and formants) for the expression of gender and gender-related attributes. However, the nature and the extent of their role have not yet been systematically investigated.

### Hypotheses

The current study explores the ability of adult speakers to alter the femininity and masculinity of their voices during an imitation experiment, as well as the extent to which they are aware of the nature of the underlying articulatory gestures that they use to make these alterations. We predict that both male and female speakers will lower their mean F0, reduce its variation, and lower their Fi, thus narrowing ΔF, when trying to sound as “masculine” as possible, whilst they will increase their mean F0 and its variation, as well as raise Fi, thus widening ΔF, to sound as “feminine” as possible. In addition, we hypothesise that speakers will round their lips in order to lengthen their vocal tract when masculinising their voice, and spread their lips to shorten their tract when feminising their voice. We also investigate male and female speakers' awareness of the contribution of F0, formant shifts and related articulatory gestures (lip/laryngeal movements) to the vocal exaggeration of masculinity and femininity.

## Materials and Methods

### Subjects

Participants were 15 female and 17 male undergraduate students from the University of Sussex (UK), between 18 and 45 years of age (M = 22.56, SD = 6.4) with no self-reported history of speech, language, or hearing disorders. All were native speakers of British English. Informed written consent was obtained for all participants before study entry.

### Procedure

Voice data were collected from individual speakers in a sound-attenuated booth at the University of Sussex. Participants were seated in a comfortable chair wearing a hat fixed to the chair in order to limit head movement, and were audio recorded with a high-fidelity microphone (AKG Perception 220).

Each participant was asked to read three different types of written stimuli out loud, first using their normal speaking voice (neutral condition), then sounding as ‘feminine’ as possible (feminine condition) and then as ‘masculine’ as possible (masculine condition), in alternate order. The material included a list of vowels embedded in a CVC context (vowel task), one short sentence that included many of the vowel sounds present in the vowel task (sentence task), and a 168 word passage comprised of several sentences (passage task – [21]). The order of presentation of the CVC words was randomized across participants to avoid serial order effects. Participants were allowed to progress at their own pace, choosing to continue to the next word only when ready. The word and sentence sequences were shown on a computer monitor, using a script written in PsyScope X Build 57. The text extract was shown in Microsoft Word 2007.

Participant's height and weight were measured prior to collecting the speech sample (Table 1). Height measurements were recorded to the nearest 0.1 cm, using a freestanding Seca Leicester stadiometer. Participants took their shoes off and stood with their shoulders flush to the stick and their heads level and oriented forward. Body weight was measured to the nearest 0.1 kg using a PS250 veterinary floor scale. Means, standard deviations and range values for participants' body size measurements are reported in Table 1.

**Table 1:** Mean, standard deviation (SD) and range values of speakers' height and weight.

	Mean	SD	Range
**Men**			
Height (cm)	181.9	6.0	171.0–188.0
Weight (Kg)	73.3	6.9	64.3–88.7
**Women**			
Height (cm)	163.3	7.1	149.6–173.6
Weight (Kg)	59.9	10.9	41.7–70.5

After completion of the vocal task, the experimenter went over a questionnaire with participants about the strategies they used to masculinise and feminise their voices, and recorded their responses on paper. The questionnaire began with a series of open questions, followed by multiple-choice questions on several vocal and articulatory gestures.

### Visual Measurements

For each participant, we measured lip spreading (LS), the horizontal distance between the two mouth corners, and openness (LO), the vertical distance between the centres of the upper and lower lips. In order to take these measurements, the horizontal mouth corners and the upper and lower centre lips were marked using a black makeup pencil (horizontal lines for the upper and lower lips, vertical lines for the mouth corners). The lip ratio for each participant was also calculated as the ratio between their lip spreading and openness. Video recordings of the participants were taken using a Sony HDR-TG3E handycam. The visual measurements were taken from stills captured using Apple iMovie version 8.0.6 of the vowel task in the neutral condition just after the participant had uttered the first consonant. Markers were then used to extract the horizontal (lip spreading) and vertical (lip openness) mouth distances using the line drawing function in Adobe Illustrator CS5.

### Acoustic Measurements

The stimuli consisted of nine monophthong British vowels in /CVC/sequences (had /æ/, head /e/, hud /Λ/, heed /i:/, hid /I/, heard /з:/, hod /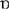/, hood /υ/, who'd /u/), the sentence “where were you a year ago?” and an extract from the “Rainbow Passage” [21]. A custom script was written in PRAAT v.5.0.3 [22] to process the collected audio samples. The script assigned a random identifier to each sample in order to ensure blind analysis. It then allowed the experimenter to set the analysis parameters and to visually compare the fundamental and formants frequencies against a narrowband spectrogram. The analysis parameters were adjusted when the computed values departed from the visually estimated fundamental and formant frequencies.

#### Fundamental Frequency

For the F0 analysis, the script used the PRAAT autocorrelation algorithm “to Pitch (ac)”, which estimates the F0 contour, from which the script derived mean F0 (F0*mean*), F0 standard deviation (F0*SD*) and the coefficient of variation (F0CV). F0*CV*, which is given by F0*SD*/F0*mean*, provides a measure of the magnitude of F0 variation relative to the mean, which reflects the logarithmic perception of pitch and therefore is a better estimate of F0 variation than its absolute estimate given by F0*SD* [17]. Perceptually, a voice with lower F0*CV* has a more monotone quality than a voice with higher F0*CV*. The parameters for F0 analysis were set as: pitch floor 30 Hz and ceiling 500 Hz for male speakers, 60 Hz and 500 Hz for female speakers, time step 0.01 s.

#### Formant Frequencies

For formant (Fi) analysis, the script used PRAAT's Linear Predictive Coding “Burg” algorithm in order to estimate the formant centre frequencies for the first four formants (F1–F4). The parameters for formant analysis were set as: number of formants 5, max formant 5000 Hz for male speakers and 5500 Hz for female speakers, and dynamic range 30 dB. The length of the analysis window was 0.025 s in the vowel and sentence tasks, and 0.5 s in the passage task.

#### Formant spacing

The centre frequencies for F1–F4 of each sample were used to calculate its average formant spacing (Δ*F*), which is the distance between any two adjacent formants:
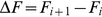
(1)Δ*F* was calculated by forcing the observed Fi values to fit the vocal tract model described in the source-filter theory [11]. In this model, the vocal tract has a uniform cross-sectional area along its entire length, which approximates the production of the vowel “schwa” (/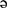/). Thus, the vocal tract acts as a quarter-wave resonator, closed at the glottis and open at the mouth, and the vocal tract resonances are given by:
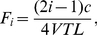
(2)where Fi is the *i^th^*-formant, c is the speed of sound in the human vocal tract (approximated to 35000 m/s) and VTL is the length of the resonator. From (1) and (2), it follows that individual formants are related to Δ*F* by:
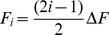
(3)Δ*F* can therefore be calculated as the slope of the linear regression expressed in equation (3), by plotting the observed F*i* (y-axis) against the expected 2*i*−1/2 formant positions (x-axis), and with the intercept set to 0 [23].

Whilst the specific variation of formants in vowels other than the “schwa” requires more complex models than the uniform quarter wavelength resonator used here [24], the average distribution of formants at suprasegmental level approaches a constant that corresponds to the ΔF predicted by such a model [7]. The adequacy of this method is illustrated by estimations of ΔF based on published acoustic data [17] presented in Figure S1. It is also consistent with perceptual observations: Smith and Patterson [25] report that Δ*F* differences re-synthesised via linear compression/expansion of the vowel spectral envelope correlate strongly with listeners' cross-class judgments of speaker's age, sex and size (man, woman, boy, girl). More recently, Pisanski and Rendall [26] also found that small (12% or 18%) uniform increments in Fi negatively correlate not only with the perceived size, but also with the masculinity of speakers within the same sex and age group.

### Statistical Analyses

Two-way mixed ANOVAs were used to investigate the overall effect of sex (group factor) and condition (as a three-level repeated factor: neutral, masculine, feminine) on each of the acoustic parameters F0*mean*, F0*CV*, Fi and Δ*F*, and on the visual parameters LS, LO and lip ratio. We also tested for differences across conditions for male and female speakers separately, running separate one-way repeated ANOVAs within each sex with condition as the factor variable and using contrasts between neutral and masculine, and neutral and feminine conditions. Levene's tests were used to check for equality of variance, and the data were log-transformed when the assumption was violated. A Mauchly's test was applied in order to check sphericity and sphericity violations were corrected for with the Greenhouse-Geisser ε. All statistical analyses were run using SPSS v.18.

## Results

The results of the ANOVAs performed on the acoustic measures are presented in Table 2 (vowel task), Table 3 (sentence task) and Table 4 (passage task). The means and standard deviations of the acoustic measures, and the F and p-values of the associated contrast are provided separately for male and female speakers in Tables 5, 6, 7 and 8.

**Table 2:** ANOVA table for the acoustic parameters in vowel task (N = 31).

Acoustic parameters	Condition		Sex		Sex×Condition	
	*F*	*p*	*F*	*p*	*F*	*p*
F0mean	55.05	<0.001*	118.75	<0.001*	1.61	0.215
F0CV	1.17	0.318	0.14	0.713	1.30	0.280
F1	10.30	<0.001*	50.58	<0.001*	5.40	0.011*
F2	25.76	<0.001*	67.50	<0.001*	2.96	0.060
F3	18.58	<0.001*	39.98	<0.001*	1.03	0.349
F4	29.27	<0.001*	60.09	<0.001*	4.78	0.024*
ΔF	30.33	<0.001*	73.13	<0.001*	2.48	0.114

F-ratio (F) and p-value (p) for: mean fundamental frequency (F0mean), coefficient of variation (F0CV), first four formant frequencies (F1–F4) and formant spacing (ΔF). Significant effects are indicated with an asterisk.

**Table 3:** ANOVA table for the acoustic parameters in sentence task (N = 32).

Acoustic parameters	Condition		Sex		Sex×Condition	
	*F*	*p*	*F*	*p*	*F*	*p*
F0mean	54.16	<0.001*	139.32	<0.001*	0.97	0.351
F0CV	3.61	0.044*	17.15	<0.001*	1.47	0.240
F1	4.73	0.018*	14.39	0.001*	6.71	0.005*
F2	14.09	<0.001*	23.92	<0.001*	1.73	0.196
F3	13.91	<0.001*	27.20	<0.001*	2.18	0.142
F4	47.71	<0.001*	72.39	<0.001*	6.15	0.011*
ΔF	41.76	<0.001*	62.28	<0.001*	2.01	0.162

F-ratio (F) and p-value (p) for: mean fundamental frequency (F0mean), coefficient of variation (F0CV), first four formant frequencies (F1–F4) and formant spacing (ΔF). Significant effects are indicated with an asterisk.

**Table 4:** ANOVA table for the acoustic parameters in passage task (N = 32).

Acoustic parameters	Condition		Sex		Sex×Condition	
	*F*	*p*	*F*	*p*	*F*	*p*
F0mean	38.26	<0.001*	186.65	<0.001*	0.69	0.506
F0CV	4.68	0.018*	4.93	0.034*	2.16	0.134
F1	13.58	<0.001*	17.83	<0.001*	4.15	0.030*
F2	17.18	<0.001*	52.56	<0.001*	1.51	0.231
F3	21.71	<0.001*	43.09	<0.001*	1.67	0.204
F4	22.73	<0.001*	88.61	<0.001*	0.52	0.561
ΔF	23.35	<0.001*	81.49	<0.001*	0.97	0.365

F-ratio (F) and p-value (p) for: mean fundamental frequency (F0mean), coefficient of variation (F0CV), first four formant frequencies (F1–F4) and formant spacing (ΔF). Significant effects are indicated with an asterisk.

**Table 5:** Mean and Standard Deviation (SD) of female speakers' acoustic parameters.

Acoustic parameters	Condition					
	Masc		Neutral		Fem	
*All vowels (N = 14)*	*mean*	*SD*	*mean*	*SD*	*mean*	*SD*
*F0mean*	185.6	25.3	202.41	22.9	256.6	55.4
*F0CV*	0.11	0.05	0.10	0.06	0.13	0.08
*F1*	568.5	59.3	648.0	92.0	667.2	90.9
*F2*	1795.8	128.8	1924.6	101.4	1948.5	109.4
*F3*	2795.6	166.9	2917.0	155.0	2964.7	121.8
*F4*	3938.6	210.3	4090.1	192.7	4123.7	153
*ΔF*	1131.1	58.9	1181.9	50.1	1195.2	43.7

*Sentence (N = 15)*						
*F0mean*	178.8	22.4	196.2	30.2	256.7	47
*F0CV*	0.19	0.10	0.25	0.10	0.21	0.08
*F1*	486.4	75.3	512.1	69.4	592.3	72.0
*F2*	1827.5	102.8	1926.4	136.2	2029.1	183.6
*F3*	2642.6	240.6	2810.6	174.3	2899.9	203.4
*F4*	3847.5	243.2	4021.7	209.6	4132.8	202.2
*ΔF*	1098	72.2	1154.7	56.3	1193.1	55.8

*Passage (N = 15)*						
*F0mean*	184.6	25.7	188.9	25.2	238.5	42.6
*F0CV*	0.18	0.10	0.23	0.10	0.23	0.10
*F1*	584.7	48.4	634.9	52.7	646	63.8
*F2*	1761.4	82.2	1831.7	93.6	1851	104.3
*F3*	2870.1	128.2	2983.9	134.2	3020.3	158.4
*F4*	3967.8	125.9	4075.2	137.1	4133.6	187.6
*ΔF*	1142.7	40.5	1180.4	44.5	1196.1	56.9

Mean and SD values (Hz) for: mean fundamental frequency (F0mean), coefficient of variation (F0CV), first four formant frequencies (F1–F4) and formant spacing (ΔF). “Masc” and “Fem” represent the masculinised and feminised conditions.

**Table 6:** Mean and Standard Deviation (SD) of male speakers' acoustic parameters.

Acoustic parameters	Condition					
	Masc		Neutral		Fem	
*All vowels (N = 17)*	*mean*	*SD*	*mean*	*SD*	*mean*	*SD*
*F0mean*	103.2	11.9	107.6	13.78	152.3	37.4
*F0CV*	0.11	0.04	0.11	0.05	0.11	0.07
*F1*	474.8	65.7	472.7	45.5	499.4	71.8
*F2*	1579.2	110.4	1619.9	88.4	1682.4	96.8
*F3*	2559.0	138.2	2609.1	126.5	2717.9	153.8
*F4*	3369.6	239.8	3508.9	236.8	3743.9	237.5
*ΔF*	990.3	58	1022.4	55	1079.6	59.9

*Sentence (N = 17)*						
*F0mean*	103.8	13.1	110.6	11.3	162.2	47.7
*F0CV*	0.10	0.06	0.15	0.10	0.2	0.05
*F1*	460.5	168.1	396.5	49.3	430.4	93.3
*F2*	1660.9	164.7	1697.9	155.4	1758.2	183,8
*F3*	2424.8	199	2436	158.8	2572.5	254.9
*F4*	3199.4	160.4	3357.2	185,3	3731.9	349.2
*ΔF*	951.5	55.5	980.3	52.9	1064.1	89.7

*Passage (N = 17)*						
*F0mean*	105.4	11.2	106	10.3	145.6	39.1
*F0CV*	0.16	0.06	0.16	0.04	0.20	0.04
*F1*	523.1	73.9	527.6	70.1	548.1	75.3
*F2*	1583.9	78.3	1606.5	65.3	1660.8	109.3
*F3*	2662.7	84.4	2701.3	64.9	2788.8	152
*F4*	3591.0	101.6	3662.2	112	3770.9	173.2
*ΔF*	1041.1	28.3	1059.3	21.6	1092.1	52.5

Mean and SD values (Hz) for: mean fundamental frequency (F0mean), coefficient of variation (F0CV), first four formant frequencies (F1–F4) and formant spacing (ΔF). “Masc” and “Fem” represent the masculinised and feminised conditions.

**Table 7:** Within-sex contrasts for the acoustic parameters across conditions in female speakers.

Acoustic parameters	Contrasts			
	Neutral vs. Masc		Neutral vs. Fem	
*All vowels (N = 14)*	*F*	*p*	*F*	*p*
*F0mean*	14.31	0.002*	24.80	<0.001*
*F0CV*	0.26	0.619	5.33	0.038*
*F1*	10.17	0.007*	0.34	0.569
*F2*	17.10	0.001*	1.59	0.229
*F3*	10.56	0.006*	2.57	0.133
*F4*	20.60	0.001*	0.99	0.002*
*ΔF*	26.17	<0.001*	2.15	0.166

*Sentence (N = 15)*				
*F0mean*	5.99	0.028*	18.26	0.001*
*F0CV*	2.49	1.370	1.622	0.224
*F1*	3.21	0.095	24.89	<0.001*
*F2*	15.83	0.001*	11.98	0.004*
*F3*	13.45	0.003*	4.58	0.050
*F4*	19.72	0.001*	6.81	0.021*
*ΔF*	32.32	<0.001*	12.32	0.003*

*Passage (N = 15)*				
*F0mean*	0.86	0.370	24.92	<0.001*
*F0CV*	6.81	0.021*	0.04	0.84
*F1*	20.23	0.001*	0.79	0.388
*F2*	13.32	0.003*	0.69	0.420
*F3*	20.96	<0.001*	1.49	0.242
*F4*	11.02	0.005*	2.08	0.172
*ΔF*	15.81	0.001*	1.78	0.210

F-ratio (F) and p-value (p) for: mean fundamental frequency (F0mean), coefficient of variation (F0CV), first four formant frequencies (F1–F4) and formant spacing (ΔF). Significant effects are indicated with an asterisk. “Masc” and “Fem” represent the masculinised and feminised conditions.

**Table 8:** Within-sex contrasts for the acoustic parameters across conditions in male speakers.

Acoustic parameters	Contrasts			
	Neutral vs. Masc		Neutral vs. Fem	
*All vowels (N = 17)*	*F*	*p*	*F*	*p*
*F0mean*	5.38	0.034*	36.95	<0.001*
*F0CV*	0.01	0.919	0.01	0.942
*F1*	0.07	0.798	4.18	0.058
*F2*	5.75	0.029*	7.08	0.017*
*F3*	7.45	0.015*	6.71	0.020*
*F4*	26.17	<0.001*	12.17	0.003*
*ΔF*	22.69	<0.001*	10.96	0.004*

*Sentence (N = 17)*				
*F0mean*	8.51	0.010*	24.33	<0.001*
*F0CV*	1.83	0.195	1.28	0.275
*F1*	2.22	0.155	1.45	0.246
*F2*	0.86	0.367	3.76	0.070
*F3*	0.17	0.688	5.93	0.027*
*F4*	20.9	<0.001*	28.3	<0.001*
*ΔF*	7.93	0.012*	23.38	<0.001*

*Passage (N = 17)*				
*F0mean*	0.84	0.776	14.48	0.002*
*F0CV*	3.12	0.096	4.11	0.060
*F1*	0.4	0.537	3.98	0.064
*F2*	6.43	0.022*	7.52	0.014*
*F3*	7.64	0.014*	7.46	0.015*
*F4*	13.46	0.002*	8.58	0.010*
*ΔF*	13.77	0.002*	8.60	0.010*

F-ratio (F) and p-value (p) for: mean fundamental frequency (F0mean), coefficient of variation (F0CV), first four formant frequencies (F1–F4) and formant spacing (ΔF). Significant effects are indicated with an asterisk. “Masc” and “Fem” represent the masculinised and feminised conditions.

### Fundamental Frequency

There was a significant main effect of sex on F0*mean* in all three reading tasks, indicating that male speakers had a lower mean F0 than female speakers across conditions, in line with the well-established sexual dimorphism in mean F0 between the two sexes.

There was also a significant main effect of condition on F0 across the three tasks. Separate ANOVAs revealed that both male and female speakers significantly raised their F0 when feminizing their voice and dropped their F0 when masculinising their voice (except when reading the passage, where the difference between neutral and masculine conditions was not significant). The largest drop in F0 between speakers' natural and masculinised voice occurred when reading the sentence, with male speakers significantly dropping their F0 by about 7% from 110.6 Hz to 103.8 Hz (Table 6) and female speakers by about 18% from 196.2 Hz to 178.8 Hz (Table 5). The smallest, yet significant, drop was recorded in reading the passage, 0.6% for men (Table 6) and 2.3% for women (Table 5). Both male and female speakers also significantly raised their F0 when feminising their voices. The largest change in F0 between speakers' natural and feminised voice occurred when reading the sentence, with male speakers raising their F0 to 162.2 Hz (about 40% rise – Table 6) and female speakers to 256.7 Hz (about 24% - Table 5), whereas the smallest, yet significant, rise was recorded in reading the passage, 28% for men (Table 6) and 20% for women (Table 5). The interaction effect between condition and sex was not significant.

### Fundamental Frequency variation (F0*CV*)

The effect of sex on F0*CV* was not significant for vowels, but was significant in the other two tasks, indicating that, overall, men spoke with a narrower dynamic range than women.

There was also a significant main effect of condition in the sentence and passage, but not for the vowels. Contrasts revealed that male speakers' F0*CV* was not significantly lower when sounding as masculine as possible than when speaking normally (although a non-significant trend was observed for the passage – Table 8). Female speakers' F0*CV* was significantly lower in the masculine condition, but only when reading the passage out loud (Table 7). There was a non-significant trend for male speakers to raise F0*CV* when reading the passage in a feminised voice (Table 8), while female speakers significantly increased their F0*CV* to feminise their voice only in the vowel task (Table 7).

### Formant frequencies

There was a significant main effect of sex on Fi in all three reading tasks indicating that male speakers' formants were lower than female speakers' across conditions.

There was also a significant main effect of condition on Fi across the three tasks. Contrasts revealed that, when asked to sound as masculine as possible, men lowered all their formants, except for F1 across conditions, F2 and F3 in the sentence task, for which no significant differences were found (Table 8). Female speakers also significantly lowered their formants when sounding as masculine as possible for all three tasks, except for F1 in the sentence task (Table 7).

When asked to sound as feminine as possible, male speakers significantly raised their formants, except for F1 across conditions and F2 in the sentence task (Table 8). Females also showed an overall tendency to raise their formants, although statistical significance was only reached for F4 in the vowel task, and F1, F2 and F4 in the sentence task (Table 7).

**Figure 1:**
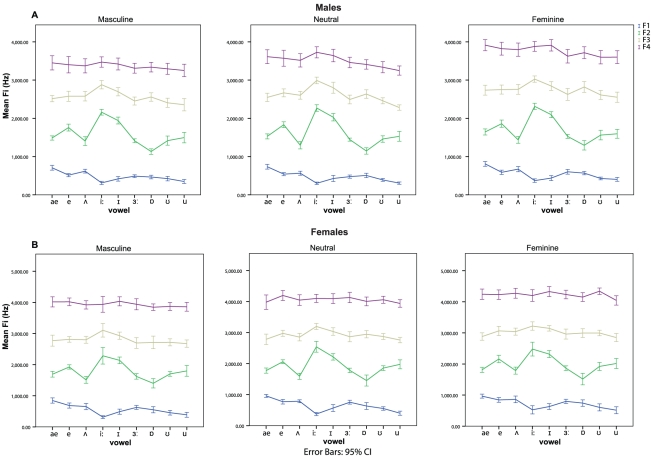
Formant values across vowels within each condition for male and female speakers. The error bar graphs show the mean (±95%CI) frequency values of the first four formant (F1–F4) across vowels and within each condition (masculine, neutral and feminine) for male (A) and female (B) speakers.

**Figure 2:**
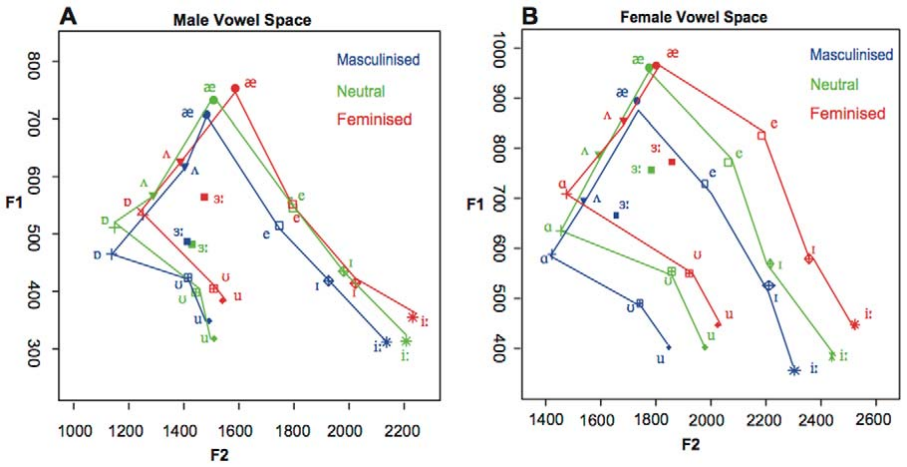
Vowel spaces of male and female speakers. Scatter plots of the mean frequency of F1 and F2 for the nine vowels spoken by men (A) and women (B) across the masculine, neutral and feminine conditions. The overall vowel spaces are outlined by joining the isolated vowels with straight lines.

Linear mixed models testing for differences in Fi were run separately for each sex as a function of condition and vowel. The results are shown graphically in Figure 1. For both men and women, there were main effects of condition and vowel on each individual formant frequency, while no significant interaction effect between condition and vowel was found on Fi (see Table 9). The vowel spaces (Figure 2) show that the vowels in the neutral condition match the typical vowel distribution in F1/F2 space for both sexes, whilst the vowel spaces in the masculine and feminine conditions match the neutral vowel space in shape, but are smaller and globally shifted downward and left, and bigger and globally shifted upward and right, respectively.

**Table 9:** ANOVA table for the vowel formant frequencies.

Women	Condition		Vowel		Condition×Vowel	
*All vowels (N = 14)*	*F*	*p*	*F*	*p*	*F*	*p*
*F1*	12.48	<0.001*	59.14	<0.001*	0.50	<0.950
*F2*	11.53	<0.001*	72.53	<0.001*	0.53	0.930
*F3*	11.99	<0.001*	12.49	<0.001*	0.48	0.960
*F4*	12.46	<0.001*	2.41	0.016*	0.68	0.811

Men	Condition		Vowel		Condition×Vowel	
*All vowels (N = 17)*	*F*	*p*	*F*	*p*	*F*	*p*
*F1*	3.53	0.03*	87.71	<0.001*	1.06	0.394
*F2*	8.26	<0.001*	178.21	<0.001*	0.65	0.841
*F3*	16.92	<0.001*	27.94	<0.001*	0.56	0.918
*F4*	50.27	<0.001*	7.36	<0.001*	0.45	0.969

Significant effects are indicated with an asterisk. “Masc” and “Fem” represent the masculinised and feminised conditions.

### Formant spacing

There was a significant main effect of sex on Δ*F* in all the three reading tasks, indicating that male speakers had a narrower overall formant spacing (Δ*F*) than female speakers. There was also a significant main effect of condition on Δ*F* across the three tasks. The interaction effect between condition and sex was not significant. Contrasts revealed that both male and female speakers significantly narrowed their Δ*F* when masculinising their voice (Tables 7 and 8). In male speakers, the extent of this decrease varied from about 2% in the passage to 3% in the other two tasks (Table 6), while in female speakers it varied from about 3% in the passage to 5% in the other two tasks (Table 5). Male speakers also significantly widened their Δ*F* when feminising their voice, and the extent of this increase ranged from 3% in the passage to 6% and 5% in the sentence and vowel tasks (Table 6), respectively, while female speakers increased their Δ*F* from 1% (passage, vowels) to 3% (sentence), reaching significance only in the sentence task.

### Lip measurements

The mean and standard deviations for the lip measurements (in pixels) taken from the vowel task in the neutral condition are presented in Table 10. The main effect of sex was significant on lip spreading (LS), F(1,21) = 8.77, p = .007, with women having a larger LS overall than men. There was also a significant main effect of condition on LS, F(2,42) = 13.86, p<.001. Contrasts revealed that both men and women significantly reduced their LS when trying to sound as masculine as possible, and increased it when sounding as feminine as possible, albeit not significantly. No significant interaction between sex and condition was found, F(2,42) = 1.39, p>.05.

**Table 10:** Mean, standard deviation (SD) and contrasts for Lip spreading (LS), Lip Openness (LO) and Lip ratio.

Women	Condition						Contrasts			
*All vowels (N = 14)*	Masc		Neutral		Fem		Neutral vs. Masc		Neutral vs. Fem	
	*mean*	*SD*	*mean*	*SD*	*mean*	*SD*	*F*	*p*	*F*	*p*
*LS*	86.5	10.7	88.4	9.6	90.7	9	5.71	0.044*	4.11	0.077
*LO*	18.7	2.0	21.1	1.5	20.5	2.2	3.94	0.082	0.29	0.603
*Lip ratio*	5.4	2	4.7	1.3	5.2	1.7	3.5	0.098	2.34	1.650

Men	Condition						Contrasts			
*All vowels (N = 17)*	Masc		Neutral		Fem		Neutral vs. Masc		Neutral vs. Fem	
	*mean*	*SD*	*mean*	*SD*	*mean*	*SD*	*F*	*p*	*F*	*p*
*LS*	66.7	17.6	69.3	19.8	69.4	18.7	6.5	0.024*	0.07	0.791
*LO*	14.6	1.2	14.5	0.9	15	1.3	.002	0.968	0.32	0.581
*Lip ratio*	5.7	1.5	5.4	1.1	5.4	1.3	.78	0.392	0.10	0.758

Significant effects are indicated with an asterisk. “Masc” and “Fem” represent the masculinised and feminised conditions.

There was a main effect of sex on lip openness (LO), F(1,21) = 7.95, p = .01, which was greater in women than in men. The main effect of condition on LO, F(2,42) = 2.08, p>.05, and the interaction effect of sex and condition, F(2,42) = 1.75, p>.05, were not significant.

As for lip ratio, the main effects of sex F(1,21) = 0.55, p>.05, condition, F(2,42) = 2.2, p>.05, and the interaction effect of condition and sex, F(2,42) = 3.71, p>.05, were all not significant.

Moreover, separate mixed model tests of differences in all three parameters were run as a function of sex, condition and vowel. There was a main effect of vowel on all three parameters (LS: F(8,535.02) = 36.35, p<.001, LO: F(8,535.17) = 57.49, p<.001, lip ratio: F(8,535.41) = 24.26, p<.001). The front vowels /æ/, /i:/, /I/, showed the highest degree of lip spreading, while lowest degree of lip spreading was recorded for the back vowels /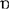/, /υ/, /u/. High vowels /υ/, /u/ also showed the least degree of lip opening, whilst low vowels exhibited the greatest lip opening. The lip ratio was smallest for vowels /æ/, /e/. There were no interaction effects between condition and vowel, and sex and vowel, indicating that both men and women moved their lips in a similar way across all three conditions.

### Participants' self-descriptions of vocal and articulatory gestures

Out of 17 male and 15 female speakers, when asked to spontaneously describe the strategies used to masculinise their voices, 9 males and 7 females replied that they made their voices sound deeper, χ^2^(32) = .13, p = .723, and 8 males and 4 females said that they made them lower, χ^2^(32) = 1.41, p = .234. To feminise their voices, 12 males and 7 females said that they made their voices higher, χ^2^(32) = 1.89, p = 1.69, and 5 males and 4 females reported making it softer, χ^2^(32) = 0.30, p = .86.

When given a choice of possible gestures, most participants reported changes in pitch: all 17 males and 14 females said that they lowered their pitch to sound more masculine, χ^2^(32) = 1.17, p = .279, and 16 males and 13 females said they raised their pitch to sound more feminine. The majority of males also reported vocal tract length adjustments: 13 males reported the descent of their Adam's apple as a gesture to masculinise their voice, compared to 6 females, χ^2^(32) = 4.39, p = .036. This was the only significant association between sex and type of strategy. Six males also reported moving their Adam's apple up to feminise their voices, compared to 4 females, χ^2^(32) = 2.76, p = .599. As for lip movements, 8 males and 11 females said they rounded their lips to sound more masculine, χ^2^(32) = 2.28, p = .131, while 8 males and 8 females said they spread their lips to sound more feminine, χ^2^(32) = 1.25, p = .723.

## Discussion

We found that when untrained adult speakers were asked to sound as masculine or as feminine as possible, they altered the frequency components of their voice (F0 and formant parameters) by adjusting the rate of vibration of their vocal folds and by changing the apparent length of their vocal tract. This shows that adult speakers have some knowledge of the sexually dimorphic acoustic cues underlying the expression of gender in speech, and are capable of controlling them to modulate gender-related attributes. Below we discuss each F0 and formant parameter individually, focusing on their acoustic and perceptual relevance in relation to previous research. Then, we compare the observed manipulations to those used to express size, and, following the “frequency code” theory [27], propose that a substantial proportion of gender-related vocal diversity in the human voice follows a “gender code”, with speakers using learned vocal gestures to manipulate their voice gender. We also look at the interplay between the observed vocal tract adjustments (e.g. lip movements and facial expressions) and the impact on gender expression. Finally, we propose some directions for future research.

### Fundamental Frequency

For both sexes, the mean F0 measured in the neutral condition was comparable to previously reported F0 values in British English [28]. The observed sex dimorphism for this parameter (1.8) is in line with previous acoustic observations [29] and can be mostly accounted for by the dimorphism in vocal fold length (1.6 – [7]). The remaining 20% of dimorphism has been attributed to sex differences in vocal fold physiology [7], [26], but may also point to differences in phonation behaviour [29], [30].

In both sexes, speakers lowered their F0 when masculinising their voices, and raised their F0 when feminising their voices, although in both conditions F0 remained within the expected range of their sex (around 100–150 Hz for men, 170–220 Hz for women – [31]). The F0 drop between the neutral and masculine conditions was about three times smaller than the F0 rise from the neutral to the feminine condition, with the smallest and non-significant drop being recorded for the passage. This could be a consequence of physiological constraints that make it more difficult for speakers to sustainably lower F0. Indeed, adult speakers speak with a mean F0 at the lower end of their physically attainable range in several languages (Traunmüller H, Eriksson A 1994 – unpublished manuscript), and this is particularly the case of male speakers of British English [28].

Perceptual studies with re-synthesised stimuli have previously reported that a F0 difference of 12% [26], [32] corresponding to twice the frequency discrimination threshold (or just-noticeable difference, JND) is required in order to elicit consistent results in discrimination performance. The observed differences in F0s between feminine/neutral and masculine/feminine conditions are above this threshold (Tables 7 and 8), suggesting that these differences are perceptually relevant. Psychoacoustic studies using natural stimuli, such as the one produced here, could confirm whether this is the case and explore the perceptual relevance of the naturally occurring acoustic variation in the vocal expression of masculinity (or femininity).

F0 variation (F0*CV*) was higher for female speakers than for male speakers in reading the sentence and the passage; these longer stimuli may enable speakers to display more intonation variation [33]. This result suggests that women speak with a wider dynamic voice range than men, which is in line with gender-stereotypes [34], but contrasts with acoustic research adopting similar log scale conversions [31], [34], [35]. In a comprehensive review of 40 years of research, Henton [31] found that previously reported male-female differences in pitch range disappeared or were reversed when re-examined using the semitonal scale (semitones = 39.86×log (F0max/F0min)). The discrepancy between the present results and Henton's may arise from the different methodologies used to model pitch perception. Although previous studies have cast doubts on the use of semitone scale as the most accurate measurement for F0 variation [36], [37], the relative value of one method over the other is yet to be established.

When asked to feminise their voices, men exhibited a non-significant trend in increasing their F0*CV* when reading the passage, but not in the other tasks. Women significantly increased their F0*CV* to feminise their voice when reading words, and decreased it to sound as masculine as possible when reading the passage. Although these differences are not consistent across all types of stimuli and between conditions, they nevertheless provide some indication that speakers may attribute wider intonation to female speech than male's, despite the fact that such attributions are largely unsupported by the literature [31]. Indeed, perceptual studies indicate that female speech is typically perceived as more ‘melodious’ than male's, both in pre-pubertal children's [38] and adults' voices [39]. Greater F0 variation also elicits higher femininity ratings, while more monotonous voices are judged to be more masculine [40].

### Formant frequencies and spacing

For both sexes, mean formant frequency values for the first four formants (F1–F4) in the neutral condition are within the range previously reported for adult speakers of Southern British English [41]–[43], with the greatest percentage difference for F1 and the smallest for F3 (F1:22.2%, F2:13.3%, F3:11.1%, F4:13.6%) between the two sexes. A similar formant scaling dimorphism was found in a study of American English [44], although their scale factors do not entirely match the present results (F1:18%, F2:17%, F3:14%).

Overall, speakers lowered their F1–F4 formants when asked to sound as masculine as possible and raised them to sound as feminine as possible. These global adjustments of formant frequency values are also reflected in the size and shifts of speakers' vowel spaces. Women's vowel space was larger and shifted top right relative to men's across conditions, in line with the known sex dimorphism [29]. However, both men and women's vowel spaces were larger, shifted upward to the right for the feminine condition, and were smaller and shifted downward to the left (Figure 2) in the masculine condition, compared to the neutral condition. This indicates that speakers exaggerated speech patterns typical of the two sexes in order to masculinise and feminise their voices.

Formant spacing (ΔF) values in the neutral condition were also comparable to those reported in the literature for both adult men (1005 Hz [45]; 991 Hz, as calculated from F1–F4 values [26]) and women (1167 Hz [26]). Moreover, men's Δ*F* was on average 15% lower than women's, in line with the ΔF dimorphism reported in previous studies [26], [46], and comparable to the 15%–20% baseline difference in anatomical vocal-tract length between the two sexes [12], [13].

Consistent with our predictions, speakers widened their Δ*F* to feminize their voices and narrowed it to masculinise them, with wider shifts in formant values being observed when imitating opposite gender attributes than when exaggerating their own gender: averaged across reading tasks, men narrowed their Δ*F* by 2.7% to masculinise their voices, whilst women widened it by 1.9% to feminise theirs, whereas men widened their Δ*F* by 5.5% to feminise their voices and women narrowed it by 4.3% to masculinise theirs. These Δ*F* differences in the expression of gender-related attributes typical of the opposite sex correspond to the limit between the male upper and female lower Δ*F* ranges [25].

Perceptually, the Δ*F* differences observed here between the natural and experimental conditions as well as between feminised and masculinised conditions (see Tables 7 and 8) are less than one JND (about 6%) for Δ*F* [29]. Thus, in combination with the percentage differences on F0 reported above, our study indicates that, although speakers adjust both F0 and Δ*F* to express gender-related attributes, only the F0 adjustments are likely to be perceived. Ultimately, by manipulating Δ*F* while preserving F0 and vice versa, future studies could look at the perceptual discriminability and relative salience of these two parameters in listeners' voice-based judgments of speakers' masculinity and femininity.

### Is there a gender code?

Indications that adjustments in F0 and Fi parameters comparable to those observed in this study play a role in the expression of voice gender and related attributes are widespread in the literature on the sex dimorphism in the human voice. Despite having virtually the same vocal anatomy, pre-pubertal boys speak with lower formants than girls [16], [17], [47], [48], suggesting that children acquire sex-specific behaviours, such as vocal tract gestures involving lip movements, to express their gender [47]. Acoustic studies of adult speakers also report within-sex differences in F0 and Fi that cannot be solely explained by anatomical differences. For example, in a cross-cultural study, Majewski [49] found that American men speak with a lower pitch (M = 118.9 Hz) than their Polish counterparts (M = 137.6 Hz), while Ohara [50] found that Japanese women raise their pitch when speaking in their native language and lower it when speaking in English, in line with femininity definitions in Japanese society. Additionally, research on the vocal expression of sexual orientation shows that, while homosexual speakers' voices do not differ in mean F0 from their heterosexual counterparts [51], [52], they display a partial shift of formant values towards those typical of the opposite sex [53], [54], even after controlling for body size [52]. Several perceptual studies also report that listeners rate adult voices characterised by higher pitch and formant values as more “feminine” [54], [55], while speakers with lower pitch and formant values are rated as more “masculine” [29], [44], [56].

These observations suggest that speakers spontaneously use a “gender code”, making a conventionalised use of the existing sex dimorphism in the frequency components of their voice to vary the expression of gender and related (e.g. masculinity/femininity) characteristics. We draw a parallel between this gender code and Ohala's [27] “frequency code” hypothesis, in which animal callers are expected to exploit the inverse correlation between resonator size and its resulting frequency in order to encode size and related (e.g. dominance/submission) attributes. Human male speakers have been shown to lower (or rise) F0 and Fi when they perceive themselves to be more (or less) dominant than their interlocutors [57], [58]. Perception studies have also reported that listeners rate speakers with lower F0 and Fi as being bigger and more dominant than speakers with higher F0 and Fi [29], [58], [59]. However, the extent to which F0 and Fi manipulations encode for both dominance and gender characteristics is yet to be systematically explored. The imitation paradigm described in this study could be used to explicitly address this question by asking speakers to express dominance and masculinity both in conjunction and separately (e.g. to sound more dominant, more masculine, dominant and masculine, dominant and feminine). Psychoacoustic studies should also investigate the perceptual relevance of F0 and Fi adjustments in gender and dominance expression and whether the same gestures are perceived differently according to speaker's and listener's personality and emotional state, situational context, semantic content and society-specific stereotypes that characterise power and gender relationships.

The present study also explored visible vocal tract length adjustments underlying the observed acoustic manipulations in formant values by providing quantitative measurements of lip movements. We found that, in line with the observed between-sex differences in overall formant spacing, lip spreading and openness were greater in women than in men in the normal voice condition, suggesting that women speak with a smile. We also found that the majority of participants perceived themselves as spreading their lips more when they feminised their voices than when speaking normally or masculinising them. In line with these self-perceptions, lip measurements revealed that speakers tended to decrease lip spreading from the feminine to the masculine conditions, although significance was only reached when speakers tried to sound as masculine as possible. In contrast, no significant differences across conditions were found for lip openness and ratio. This suggests that lip gestures alone cannot fully account for the observed formant shifts. Indeed, while it was not possible to track vertical laryngeal displacement, more than one third of the participants, and particularly men, reported moving their larynx along the existing sex dimorphism in the experimental conditions and especially when masculinising their voices. It is possible that the enhanced protrusion of the human male larynx, compared to the female larynx, allows male speakers to be more aware of any movement in its position. It is worth noting that the males of several other mammalian species are known to actively lower their larynges during vocalisation in order to extend their vocal tracts and thus exaggerate the vocal expression of their body size (red deer [60], fallow deer [61]), pointing at selection pressures underlying the sexual dimorphism of the vocal tract (deer [62], humans [14]). A recent study also indicates that vocal tract length adjustments affect attributions of physical and social dominance in human males [58].

Further investigations should consider more sophisticated techniques to better quantify lip movements (e.g. motion tracking [63], [64]), as well as measure laryngeal vertical shifts (e.g. using ultrasound or MRI) in order to establish the respective role of such adjustments in the manipulation of vocal tract length to vary the expression of gender or related attributes.

Finally, the observed lip gestures performed to feminise or masculinise the apparent gender of the voice are likely to impact facial expressions and associated gender stereotypes. While Ohala [27] suggested that the retraction of lip corners to sound smaller and their rounding and protrusion to sound bigger are, respectively, at the origin of the smile and the “o-face” which are common in dominance displays, we propose that individuals feminising their voice are likely to spread their lips, and therefore project a “cheerful”, unthreatening face, and those masculinising their voice are likely to round their lips, and therefore project a more “angry”, dominant face. Indeed, women tend to smile more than men [65], possibly following cultural norms [66]–[69].

### Future directions

The present study shows that untrained speakers have the spontaneous ability to modify the expression of their gender and related traits through the voice, but does not shed light on their acquisition and use in every day life. We suggest that future studies could (i) extend the imitation paradigm adopted in this study to children and investigate the acquisition and development of sex-typical ways of speaking according to age, (ii) investigate whether children and adults vary the expression of their gender in different settings, and when complying with varying gendered and sex roles within and across different societies, as well as the perceptual relevance of these variations.

## Supporting Information

Figure S1 **Illustration of the fitness of the method used to estimate overall formant spacing.** Frequency values of F1,F2 and F3 for male (A) and female (B) adult (>19 years old) speakers as measured in Lee et al. [17] plotted against (2i−1)/2 increments of the formant spacing as predicted by a uniform vocal tract model. Formant spacing ΔF can be estimated as the slope of the linear regression of observed Fi over the expected formant positions (with intercept set to 0). The apparent Vocal Tract Length (aVTL expressed in centimetres) can be calculated as aVTL = c/2ΔF. The values of Δ*F* reported in the figures correspond to aVTL values of 17.71 cm for male speakers and 14.95 cm for female speakers, which are comparable to anatomical vocal tract lengths in adult men and women (men: 18 cm, women: 15 cm [10]). This illustrates that, while Δ*F* estimated in this way is sensitive to vowel-specific variation in vocal tract configuration, at supra-segmental level it provides an estimate of the overall linear scaling of the formants which is a reliable estimate of the average vocal tract length of the speaker.
(TIF)
