# Supplementary figures and images for: Spontaneous Voice Gender Imitation Abilities in Adult Speakers
Source: PLoS One. 2012 Feb 17;7(2):e31353. doi: 10.1371/journal.pone.0031353 (PMC3281965; doi:10.1371/journal.pone.0031353)

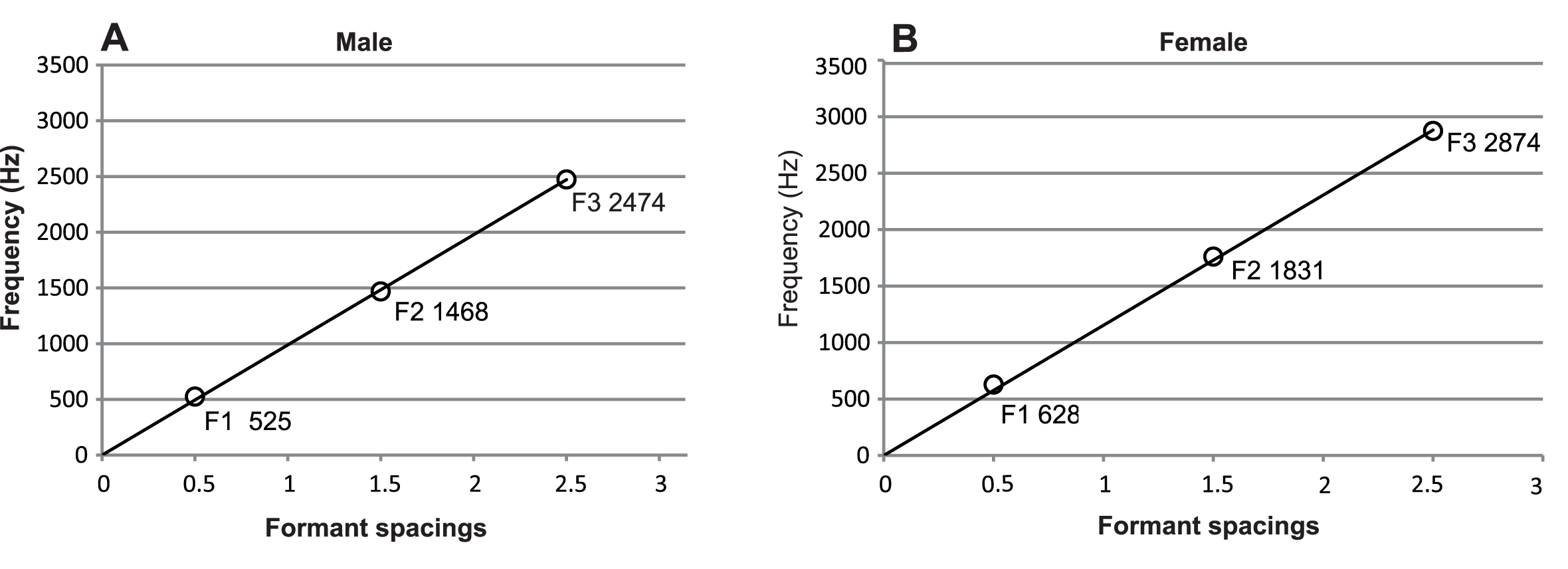

Supplement: Figure S1 — Illustration of the fitness of the method used to estimate overall formant spacing. Frequency values of F1,F2 and F3 for male (A) and female (B) adult (>19 years old) speakers as measured in Lee et al. [17] plotted against (2i−1)/2 increments of the formant spacing as predicted by a uniform vocal tract model. Formant spacing ΔF can be estimated as the slope of the linear regression of observed Fi over the expected formant positions (with intercept set to 0). The apparent Vocal Tract Length (aVTL expressed in centimetres) can be calculated as aVTL = c/2ΔF. The values of ΔF reported in the figures correspond to aVTL values of 17.71 cm for male speakers and 14.95 cm for female speakers, which are comparable to anatomical vocal tract lengths in adult men and women (men: 18 cm, women: 15 cm [10]). This illustrates that, while ΔF estimated in this way is sensitive to vowel-specific variation in vocal tract configuration, at supra-segmental level it provides an estimate of the overall linear scaling of the formants which is a reliable estimate of the average vocal tract length of the speaker. (TIF) [file pone.0031353.s001.tif]
